# Aliskiren, Fosinopril, and Their Outcome on Renin-Angiotensin-Aldosterone System (RAAS) in Rats with Thyroid Dysfunction

**DOI:** 10.1155/2019/5960563

**Published:** 2019-07-18

**Authors:** Susan A. S. Farhadi, Kawa F. Dizaye

**Affiliations:** Department of Basic Sciences/ Pharmacology Unit, College of Medicine, Hawler Medical University, Erbil, Iraq

## Abstract

**Background and Objectives:**

Thyroid hormones have an important role in the growth and development of various tissues including the kidney, which is the major site of renin release and the consequent angiotensin and aldosterone formation. Therefore any derangement in thyroid function can result in abnormal functioning in the renin-angiotensin-aldosterone system. The current study was undertaken to find the impact of using a direct renin inhibitor (Aliskiren) and an angiotensin-converting enzyme inhibitor (Fosinopril) on the components of the renin-angiotensin-aldosterone system (RAAS) in rats with thyroid dysfunctions.

**Method:**

Forty-two male albino rats were divided into three subgroups. First group (6 rats) served as control. Second group (18 rats) served as hyperthyroid group (6 rats positive control, 6 rats given Aliskiren, and 6 rats given Fosinopril). Third group (18 rats) served as hypothyroid group (6 rats positive control, 6 rats given Aliskiren, and 6 rats given Fosinopril). Induction of hyperthyroidism and hypothyroidism was done through daily oral administration of L-Thyroxine and Propylthiouracil, respectively. On day 40 of the study, the rats were sacrificed and blood was collected for estimation of renin, angiotensin I, angiotensin II, aldosterone, TSH, T_3_, and T_4_. The collected blood samples were also used for estimation of levels blood urea, serum creatinine, liver enzymes, and serum electrolytes. Blood pressure and urine collection were done on days 1 and 40. The collected urine was used for estimation of urine flow, sodium excretion, and potassium excretion rates.

**Results:**

In hypothyroid induced rats, serum renin level dropped as expected, while the use of Aliskiren and Fosinopril on these hypothyroid rats raised renin level due to the feedback mechanism. Both angiotensin I and II were significantly (P <0.05) lower than normal levels in the hypothyroid rats, unlike the level of aldosterone, which was higher than normal level. There was nonsignificant lowering in BP (systolic, diastolic, and mean BP) in the hypothyroid rats. Treatment of these rats with Aliskiren and Fosinopril did not lower the blood pressure more than normal when compared to the hypothyroid group. The hypothyroid rats also showed a decrease in level of serum creatinine. In hyperthyroid rats, there was a rise in levels of serum renin, angiotensin II, and aldosterone; nevertheless, the increase in angiotensin I level was significant. The use of Aliskiren and Fosinopril increased the level of renin nonsignificantly (decreased angiotensin I significantly). Hyperthyroid rats showed a significant increase in systolic, diastolic, and mean blood pressure. Both Aliskiren and Fosinopril increased urine flow, Na^+^ excretion, and K^+^ excretion rates. Aliskiren was better at reducing the high blood pressure.

**Conclusion:**

Aliskiren and Fosinopril in hyperthyroid rats decreased serum angiotensin I, angiotensin II, and aldosterone. Blockade of renin and inhibition of angiotensin-converting enzyme both resulted in a rebound increase in level of renin in hypothyroid rats. Aliskiren is better at controlling blood pressure in hyperthyroid rats. Urine flow, sodium excretion, and potassium excretion rates were improved by the use of Aliskiren and Fosinopril in hyperthyroid rats.

## 1. Introduction

The thyroid gland has one of the most important functions in the human body as it regulates most of the body's physiological actions. It produces hormones (tetraiodo-L-thyronine (T4) and triiodo-L-thyronine (T3)) that are responsible for many actions including metabolism, development, protein synthesis, and the regulation of many other important hormones and enzymes [1]. Abnormal functioning of the thyroid can affect the production of thyroid hormones (T3 and T4) which could be linked to many pathologies throughout the body [2]. Most of T4 is converted to T3 by 50-monodeiodination in the liver, kidneys, and skeletal muscle; therefor T4 is regarded as a prohormone [3].

Thyroid hormone disorders, whether hypofunction or hyperfunction, can have essential effects on the cardiovascular system [4].

Thyroid hormones have direct and indirect effects on the cardiovascular function, through effects on the heart and effects on the peripheral vasculature, respectively [5]. Any alterations in thyroid functions (hypothyroidism and hyperthyroidism) might disrupt the renal and cardiovascular functions [6].

Hyperthyroidism leads to a hypermetabolic state that is characterized by increased resting energy expenditure, increased weight loss, decreased cholesterol levels, increased lipolysis, and gluconeogenesis [7].

Deficiency of thyroid hormone leads to a clinical state of hypothyroidism. The deficiency could be due to reduced production, deranged distribution, or lack of thyroid hormone effects. It is characterized by decreased metabolic rate and by a high serum thyroid-stimulating hormone [8].

Thyroid hormones have important effects at cardiac and vascular level. They directly affect heart rate control, myocardial excitability, inotropic status, systemic vascular resistance, and blood pressure. Furthermore, they can affect cardiovascular system activity by interacting with other factors (catecholamines, renin-angiotensin-aldosterone system) [9].

One of the most important mechanisms that regulate blood pressure, fluid volume, and sodium-potassium balance is the renin-angiotensin-aldosterone system (RAAS). That is why change in any molecules that compose RAAS contributes to developing arterial hypertension [10].

Renin and angiotensin-converting enzyme (ACE) are part of the renin-angiotensin-aldosterone system (RAAS) and are important regulators of blood pressure and salt-water homeostasis [11].

Aliskiren is a direct inhibitor of the enzyme renin and it is indicated for the treatment of essential hypertension. It can be used alone or in combination with other antihypertensive agents for the treatment of high blood pressure [12]. Aliskiren acts on the renin-angiotensin-aldosterone system, by inhibiting the production of angiotensin I and II with a resultant reduced plasma renin activity [13]. Aliskiren has demonstrated renoprotective effects in experimental animal models such as renal ischemia/reperfusion injury [14].

Angiotensin-converting enzyme inhibitors (ACEIs) are a group of drugs that are effective in controlling high blood pressure and their use in patients has been linked to reduced cardiovascular morbidity and mortality [15].

Fosinopril is an angiotensin-converting enzyme inhibitor that has great effect on reducing blood pressure [16]. It acts as a competitive inhibitor of angiotensin-converting enzyme and consequently prevents the conversion of angiotensin I to angiotensin II. By doing so, Fosinopril significantly lowers systemic vascular resistance, lowers blood pressure, and improves cardiac function [17]

Fosinopril is a prodrug, which is hydrolyzed to pharmacologically active metabolite, Fosinopril, in liver and gastrointestinal mucosa [18].

Both hypothyroidism and hyperthyroidism can cause renal and cardiovascular dysfunction [19]. Thyroid hormones increase the tissue response to the action of the sympathetic nervous system, and this may be a mechanism by which they regulate BP. Thyroid hormones have an important role in the growth and development of various tissues, including the kidney and lung, which are major sites of renin and ACE synthesis; therefore, thyroid hormone deficiency in early developmental stages can have significant effects on RAS components [20].

This study was conducted to find out the various effects of angiotensin-converting enzyme inhibitors (ACE inhibitors) (Fosinopril) and direct renin inhibitors (Aliskiren) on the RAAS system in rats with thyroid disorders.

## 2. Methods

### 2.1. Animals

Laboratory male albino rats with body weight 200–320 grams were used in the present study. Rats were kept in cages in the animal house of College of Medicine (Hawler Medical University). They were housed under controlled conditions of illumination (12 h light/12 h darkness) and temperature 20°C–25°C, throughout the experimental period. They had free access to standard pellet diet fortified with vitamins and water.

### 2.2. Materials

Elabscience ELISA (Enzyme-Linked Immunosorbent Assay) rat kits were used for TSH, renin, angiotensin I, angiotensin II, and aldosterone analysis. Fosinopril sodium (Monopril) 20 mg manufactured by Deva was purchased from a licensed pharmacy in Istanbul, Turkey. Aliskiren (Rasilez) 150 mg manufactured by Novartis was purchased from Germany. Propylthiouracil (Prouracil) 50 mg manufactured by Iran Hormone was used for induction of hypothyroidism. Levothyroxine (Eltroxin) 100 mcg manufactured by aspen was used for hyperthyroid induction.

### 2.3. Study Design

Forty-two rats were used in this current study. The rats were divided into three groups. The first group of 6 rats served as a control. The second group was of 18 hypothyroid rats, which were subdivided into three subgroups (each subgroup consisted of 6 rats). The first subgroup served as positive control. The second and third subgroups received a daily dose (10 mg/kg) of Fosinopril and Aliskiren, respectively [21]. The drugs were delivered orally by gastric gavage at the same time each morning.

The third group involved 18 hyperthyroid rats, which were divided into three subgroups (each of 6 rats). Again the first subgroup served as positive control. The second and third subgroups received a daily dose (10 mg/kg) of Fosinopril and Aliskiren, respectively [21]. The drugs were delivered orally by gastric gavage at the same time each morning.

Blood pressure, heart rate, body weight, and 24-hour urine volume were recorded on the first day of the study and were repeated on the 40^th^ day. Daily Aliskiren and Fosinopril were given in the last 14 days of the study. Blood pressure and heart rates were recorded using noninvasive CODA monitor. The rats were put in a specialized cage and blood pressure was recorded through a tail cuff. For each rat 5 readings of blood pressure were recorded. Urine collection was done through the use of a metabolic cage and it was used for estimation of urine flow rate, sodium excretion rate, and potassium excretion rate.

The hyperthyroid groups were given (0.0012% w/v) L-Thyroxine in drinking water for 40 days [22] and the hypothyroid groups were given Propylthiouracil (PTU) (0.05% w/v) [23].

On the 40^th^ day of the experiment, the animals were anaesthetized with an intraperitoneal injection of Xylazine 5 mg/kg and Ketamine 35 mg/kg [24]. Blood was withdrawn through a cardiac puncture. The collected blood was centrifuged then the serum was used for serologic tests like levels of renin, angiotensin I & II, aldosterone, TSH, and total T_3_ & T_4_ and biochemical tests like blood urea, serum creatinine, and liver function tests.

### 2.4. Ethical Committee

This study was approved by the Ethical Committee of College of Medicine, Hawler Medical University, in the meeting coded 6, paper code 3 dated 23^rd^ of April 2016.

### 2.5. Statistical Analysis

Data was analyzed statistically using the Statistical Package for the Social Sciences (SPSS) Version 20.0 for Windows. All the data was expressed as mean ± SD and SE. Comparisons between groups were done using Duncan test and student t-test. P value of 0.05 or less was considered statistically significant

## 3. Results

Induction of hyperthyroidism in rats by using daily L-Thyroxine significantly (P <0.05) increased the level of T_4_ when compared to normal rats, while the change in T_3_ level, although higher than normal, was statistically nonsignificant.

TSH on the other hand showed a significant drop in its level compared to the control rats (Table 1)

Daily PTU administration for induction of hypothyroidism significantly (P <0.05) lowered levels of both T_3_ and T_4_ while TSH level was profoundly higher than the level in normal rats (Table 1).

Hyperthyroid rats showed a nonsignificant increase of renin level. Although this parameter was increased in both Fosinopril and Aliskiren groups, the changes were nonsignificant when compared to the hyperthyroid group (Table 2).

On the other hand, angiotensin I was significantly increased in hyperthyroid group compared to the control group. Both Aliskiren and Fosinopril caused a significant decrease (P <0.05) (Table 2).

The effect of hyperthyroidism on angiotensin II was a nonsignificant increase, while Aliskiren and Fosinopril caused significant lowering when compared to the hyperthyroid group (Table 2).

Aldosterone was slightly and nonsignificantly increased in hyperthyroid group while both Aliskiren and Fosinopril caused a significant reduction in the serum level of aldosterone in hyperthyroid rats (Table 2).

Although hypothyroidism markedly lowered serum renin, the change was nonsignificant while it was significantly increased (P <0.05) in Fosinopril and Aliskiren groups when compared to the hypothyroid group and normal rats (Table 3).

Compared to the control group, hypothyroid induced a significant reduction of serum angiotensin I, but the change was nonsignificant in Fosinopril and Aliskiren groups when compared to the hypothyroid group (Table 3).

Table 3 shows that angiotensin II was nonsignificantly reduced in hypothyroid, Fosinopril, and Aliskiren groups.

Hypothyroidism nonsignificantly increased the level of aldosterone while this parameter was markedly but statistically nonsignificantly reduced in Fosinopril and Aliskiren groups when compared to the hypothyroid group (Table 3).

Following induction of hyperthyroidism in the rats, systolic BP was significantly increased (P <0.05). There was a significant reduction in Aliskiren group while the reduction was nonsignificant by Fosinopril compared to the hyperthyroid group (Table 4).

Compared to normal rats, the hyperthyroid rats showed a significant rise in Diastolic BP. When hyperthyroid rats were treated with Aliskiren, there was a significant reduction of this parameter but the change by Fosinopril was nonsignificant (Table 4).

In Table 4 it can be seen that hyperthyroidism induced by daily Thyroxine significantly increased mean BP and Aliskiren significantly reduced the parameter, but the reduction caused by Fosinopril was irrelevant.

The changes in heart rate were negligible in all groups (Table 4).

Hypothyroid induction by PTU resulted in a nonsignificant reduction of systolic, diastolic, and mean BP. The same results were observed in Aliskiren and Fosinopril treated groups (Table 5).

While heart rate was significantly reduced in the hypothyroid group, it was nonsignificantly reduced in Aliskiren and Fosinopril groups compared to the hypothyroid group (Table 5).

This table shows the results of blood urea, serum creatinine, and liver function enzymes in normal, hyperthyroid, and hyperthyroid groups treated with Aliskiren and Fosinopril. Urea was significantly reduced in the hyperthyroid rats. Both Aliskiren and Fosinopril were able to reduce urea levels too, but the reduction was nonsignificant (Table 6).

Neither hyperthyroidism nor treatment with Aliskiren and Fosinopril had any effect on serum creatinine (Table 6).

The rise in AST level in hyperthyroid and Aliskiren groups was nonsignificant. Although the Fosinopril group shows a marked decrease in AST, the change was nonsignificant (Table 6).

The changes that were observed in ALT and ALP levels were nonsignificant in all groups (Table 6).

No significant changes were observed in blood urea, serum creatinine, and liver function tests of all groups (Table 7).

Although Aliskiren was able to cause twofold increase in the rate of urine flow in hyperthyroid rats, it was nonsignificant. It was noticed that Fosinopril has a greater effect in increasing urine flow rate, as Fosinopril has caused significant threefold increase (P <0.05) in urine flow when compared to the hyperthyroid group (Table 8).

While there was a nonsignificant change in the sodium excretion rate of the hyperthyroid group, the rise of this parameter in the Aliskiren and Fosinopril treated groups was significant (Table 8).

Hyperthyroidism nonsignificantly reduced potassium excretion rate. However it was significantly increased (P <0.05) by daily administration of Aliskiren and Fosinopril to the hyperthyroid rats (Table 8).

The reduction in urine flow is negligible in the hypothyroid group as well as the Fosinopril treated group. While in Aliskiren treated group urine flow was increased, this was nonsignificant when compared to the hypothyroid group (Table 9).

Table 9 reveals that the rate of sodium excretion increased but nonsignificantly in the hypothyroid and Aliskiren groups while it was nonsignificantly reduced in the Fosinopril treated group when compared to the hypothyroid group.

Both hypothyroidism and Aliskiren treated groups showed a nonsignificant rise in rate of potassium excretion, while Fosinopril on the other hand caused a significant reduction in the rate when compared to the hypothyroid group (Table 9).

In Table 10 it was observed that Fosinopril was able to decrease the level of serum sodium in hyperthyroid rats significantly while the changes in hyperthyroid and Aliskiren groups were irrelevant.

Although Aliskiren and Fosinopril treated groups showed an increase in serum sodium levels of hypothyroid rats, the changes were within the normal range and were regarded as nonsignificant (Table 10).

On the other hand, no significant changes were seen in the serum potassium level of all groups (Table 10).

In Table 11 it can be observed that there were no significant changes in the weights of rats during induction of hyperthyroid and hypothyroid states and after the groups were treated with Aliskiren and Fosinopril.

## 4. Discussion

The results obtained from this study revealed that induction of hyperthyroidism with the daily use of L-Thyroxine significantly increased the level of T_4_ and lowered TSH level significantly when they were compared to the normal rats. Thyroxine is a synthetic T_4_ and is identical to the natural hormone in its action; as a result, there will be a rise in levels of T_3_ and T_4_ and a drop in TSH level due to hypothalamic pituitary feedback mechanism. On the other hand, hypothyroid induction by daily PTU resulted in a significant drop in levels of circulating T_3_ and T_4_ with a significant rise in TSH level compared to the euthyroid rats. PTU acts by inhibiting the iodination of tyrosyl residues in thyroglobulin along with inhibition of peripheral conversion of T_4_ to T_3._ Through the negative feedback mechanism, levels of TSH will be increased [25].

It is well known that thyroid hormones regulate BP by increasing the response of cardiovascular system to the action of the sympathetic nervous system or/and by directly activating the RAAS [26].

In this study we have observed that hyperthyroidism has caused an upsurge in the levels of serum renin, angiotensin II, and aldosterone, but the changes were of no significance when they were compared to the normal rats. On the other hand, angiotensin I raise was of statistical significance in the hyperthyroid induced rats. These results are in accordance with that of Ramos et al. (2006) [27] indicating that the RAAS components increase in hyperthyroidism. The results obtained from treatment of hyperthyroid rats with daily Aliskiren and Fosinopril were a one- to twofold increase, respectively, in the level of serum renin, but this change could be attributed to the initial inhibition of RAAS which is usually associated with a rebound increase through feedback mechanisms. This is in accordance with the work of Batenburg et al. (2008) [28] which showed that renin inhibitors, like all inhibitors of renin-angiotensin system (RAS), increase the concentration of renin because they enhance the negative feedback effect of angiotensin II on renin release. Angiotensin I, angiotensin II, and aldosterone were lowered in both groups treated with Aliskiren and Fosinopril. It has been pointed out by Pool (2007) and Atlas (2007) that inhibition of renin and ACE inhibitors decrease levels of angiotensin I, angiotensin II, and aldosterone [29, 30].

In the hyperthyroid rats, systolic, diastolic, and mean blood pressure were obviously increased and these changes are the consequences of sympathetic overactivity, which is characteristic of hyperthyroidism just as has been observed by Chen et al. (2010) [31] that hyperthyroidism is characterized by clinical manifestations that resemble the state of increased adrenergic activity. Furthermore, the fact that beta-adrenergic receptor blockers improve these symptoms and signs has again suggested activated sympathetic nervous system in hyperthyroidism. While the changes in blood pressure were of significance, the increase in heart rate although obvious was of no statistical importance. Compared to the hyperthyroid group, the two groups treated with Aliskiren and Fosinopril managed to reduce systolic, diastolic, and mean BP, but the changes were more prominent in the Aliskiren treated hyperthyroid group, which is consistent with the study of Drummond et al. (2007) [32] on Aliskiren that shows a marked reduction in BP upon the use of this drug in hypertensive patients. Aliskiren's inhibition of renin and the subsequent formation of angiotensin I and angiotensin II lead to better control over blood pressure. According to the results of this study, we can suggest that Aliskiren is the preferable drug for lowering high blood pressure in hyperthyroid patients especially those with systolic hypertension like in elderly patients and in hypertensive patients with atherosclerosis as Aliskiren indirectly affects blood volume (by increasing urine flow and reducing aldosterone formation), which results in a reduction in cardiac output and consequently lowering systolic blood pressure. It is well known that hyperthyroid is associated with increased cardiac contractility and cardiac output [33]. Therefore, the use of Aliskiren will be a good choice in treating hypertension in patients with hyperthyroidism

The results obtained from this study show that hyperthyroidism reduced levels of blood urea. Urine flow in the hyperthyroid rats was slightly decreased but their medication with Aliskiren and Fosinopril caused two- and three-fold increase in the rate, respectively. Along with the urine flow, Na^+^ and K^+^ excretion rates were markedly increased, which is consistent with the work of Nussberger et al. (2002) [34] who have concluded that Aliskiren has natriuretic effects.

In the current study the results show, except for serum sodium in Fosinopril treated group, that Fosinopril decreases the formation of angiotensin II and consequently aldosterone release. Aldosterone is known for its salt and water retaining properties. As Fosinopril decreases serum sodium levels, it is preferable for patients with salt sensitive hypertension. The effects of hypothyroidism on body functions are usually opposite to the effects of hyperthyroidism. As was concluded by Kumar et al. (2013) [35], thyroid hormones have a role in the maturation of the RAAS system; therefore in hypothyroidism plasma level of renin is usually low. In the current study although the result obtained was a marked drop in renin level, it was of no statistical significance when compared to the normal rats. The hypothyroid rats treated with Aliskiren and Fosinopril revealed a significant rise in the level of serum renin, which could be attributed to the initial rise of renin following the use of a direct renin inhibitor and an ACE inhibitor leading to a rebound increase due to enhancement of negative feedback mechanisms as mentioned earlier [28].

In hypothyroidism there was significant lowering in angiotensin I.

Induction of hypothyroidism by the use of daily PTU lowered systolic, diastolic, and mean BP nonsignificantly when compared to the euthyroid rats and such a result is expected, as there will be a decrease in sympathetic activity in hypothyroidism. This lowering in BP is in contrast to other studies which refer to long term hypothyroidism resulting in an increase in BP mainly diastolic and this is due to development of atherosclerosis, but this study showed a decrease in blood pressure which could be related to the duration of the study which lasted for only 40 days. Administration of Aliskiren and Fosinopril to the hypothyroid rats did not have a significant effect on the above parameters. While the changes in BP were minimal, the effect of hypothyroidism on heart rate was a marked drop in the rate compared to the normal rats, as hypothyroidism is characterized by decreased sympathetic activity and dominance of vagal stimulation with its accompanying bradycardia.

The result obtained from this study on effect of hypothyroidism on the level of serum creatinine was a marked but nonsignificant rise when compared to the normal rats and this is in accordance with the results of Kaur et al. ( 2015) [36] who have concluded that there was a significant rise in serum creatinine in hypothyroid state and that there is a correlation between the rise in TSH levels and serum creatinine levels.

Potassium excretion rate in hypothyroid rats treated with Fosinopril showed significant lowering when compared to the hypothyroid rats, as Fosinopril, like other renin-angiotensin inhibitors, decreases the action of aldosterone. Aldosterone is a well-known enhancer of renal potassium excretion. Therefore its inhibition results in decreased renal excretion of potassium.

Induction of hypothyroidism and treatment of the hypothyroid rats with Aliskiren and Fosinopril showed no significant changes in the levels of serum sodium and potassium when compared to the hypothyroid group. Physiologically, serum sodium concentration is maintained constant due to effect of different factors like kidney function, sympathetic nervous activity, and hormones (e.g., natriuretic peptides) [37].

## 5. Conclusions

Aliskiren and Fosinopril in hyperthyroid rats decreased serum angiotensin I, angiotensin II, and aldosterone. Blockade of renin and inhibition of angiotensin-converting enzyme both resulted in a rebound increase in level of renin in hypothyroid rats.

Aliskiren is better at controlling blood pressure in hyperthyroid rats.

Urine flow, sodium excretion, and potassium excretion rates were improved by the use of Aliskiren and Fosinopril in hyperthyroid rats, but in hypothyroid rats Fosinopril has decreased potassium excretion rate.

Fosinopril decreased serum Na^+^ in hyperthyroid rats.

## Figures and Tables

**Table 1:** Mean and SE (standard error) of serum levels of T_3_ (tri-iodothyronine), T_4_ (thyroxine), and TSH (thyroid stimulating hormone) in normal, hyperthyroid, and hypothyroid rats.

	Normal Control	Hyperthyroid	Hypothyroid
T_3_ nmol/L	1.73±0.04	2.33±0.29	1.03±0.08 *∗∗*

T_4_ *μ*g/dL	78.88±16	295.62±16.15*∗∗*	17.52±4.37 *∗∗*

TSH ng/L	6.08±0.89	1.21±0.25 *∗∗*	19.55±4.26 *∗*

*∗∗* Compared to control, P<0.001.

*∗* Compared to control, P<0.05.

**Table 2:** Effects of hyperthyroidism, Aliskiren, and Fosinopril on serum levels of renin, angiotensin I & II, and aldosterone.

Data	Control	Hyperthyroid	Aliskiren	Fosinopril
Renin	274.57±24	372.42±42	531.44±129	462.05±52
pg/mL	a	ab	b	ab

Angiotensin I	216.22±5	244.90±9	207.92±4	187.86±5
pg/mL	b	a	b	c

Angiotensin II	143.25±9	166.24±4	119.16±20	114.08±4
pg/mL	ab	b	a	a

Aldosterone	353.86±36	363.10±27	273.32±16	245.00±16
pg/mL	a	a	b	b

Different letters indicate significance differences at P<0.05.

**Table 3:** Effects of hypothyroidism, Aliskiren, and Fosinopril on serum levels of renin, angiotensin I & II, and aldosterone.

Data	Control	Hypothyroid	Aliskiren	Fosinopril
Renin	274.58±24	191.30±19	779.28±126	462.05±52
pg/mL	a	a	b	b

Angiotensin I	216.23±5	181.012±12	182.01±5	187.86±5
pg/mL	a	b	b	b

Angiotensin II	143.25±9	109.16±9	124.23±6	134.86±15
pg/mL	a	a	a	a

Aldosterone	353.86±6	396.16±49	264.40±15	306.88±43
pg/mL	a	a	a	a

Different letters indicate significance differences at P<0.05.

**Table 4:** The relation between blood pressure (BP) and heart rate in control, hyperthyroid, Aliskiren, and Fosinopril groups.

	Control	Hyperthyroid	Aliskiren	Fosinopril
Systolic BP	119.38±2	147.77±3	121.72±3	136.14±4
mmHg	a	b	a	b

Diastolic BP	88.57±1	107.33±4	88.65±3	98.78±2
mmHg	a	b	a	ab

Mean BP	98.46±1	120.83±4	99.50±3	111.00±2
mmHg	a	b	a	ab

Heart rate	382.53±7	425.26±19	414.23±16	390.64±11
Beat/Minute	a	a	a	a

Different letters indicate significance differences at P<0.05.

**Table 5:** The relation between blood pressure (BP) and heart rate in control, hypothyroid, Aliskiren, and Fosinopril groups.

	Control	Hypothyroid	Aliskiren	Fosinopril
Systolic BP	119.38±2	107.07±4	107.58±2	105.83±5
mmHg	a	a	a	a

Diastolic BP	88.57±1	80.83±4	81.85±3	79.81±4
mmHg	a	a	a	a

Mean BP	98.46±1	89.35±4	90.17±3	88.33±5
mmHg	a	a	a	a

Heart rate	382.53±7	301.93±3	312.10±4	298.63±2
Beat/Minute	a	b	b	b

Different letters indicate significance differences at P<0.05.

**Table 6:** Blood biochemistry tests in control, hyperthyroid, Aliskiren, and Fosinopril groups.

	Control	Hyperthyroid	Aliskiren	Fosinopril
Urea	58.22±7	40.20±1	33.40±1	33.00±1
mg/dl	a	b	b	b

Creatinine	0.451±0.04	0.452±0.009	0.446±0.01	0.446±0.01
mg/dl	a	a	a	a

AST	125.65±34	134.40±8	146.80±18	104.20±7
unit/L	a	a	a	a

ALT	45.32±6	51.20±4	49.20±5	43.60±2
unit/L	a	a	a	a

ALP	228.09±88	256.40±41	190.80±27	174.80±16
unit/L	a	a	a	a

Different letters indicate significance differences at P<0.05.

Abbreviations. AST: aspartate aminotransferase, ALT: alanine aminotransferase, ALP: alkaline phosphatase.

**Table 7:** Blood biochemistry tests in control, hypothyroid, Aliskiren, and Fosinopril groups.

	Control	Hypothyroid	Aliskiren	Fosinopril
Urea	58.22±7	49.72±3	34.60±5	68.80±27
mg/dl	a	a	a	a

Creatinine	0.45±0.04	0.69±0.05	0.48±0.04	0.65±0.14
mg/dl	a	a	a	a

AST	125.65±34	147.00±22	126.60±13	129.60±8
unit/L	a	a	a	a

ALT	45.32±6	41.83±7	59.20±4	52.80±4
unit/L	a	a	a	a

ALP	228.09±88	262.95±73	197.60±18	257.60±34
unit/L	a	a	a	a

Different letters indicate significance differences at P<0.05.

Abbreviations. AST: aspartate aminotransferase, ALT: alanine aminotransferase, ALP: alkaline phosphatase.

**Table 8:** Urine flow, Na^+^ excretion rate, and K^+^ excretion rate in hyperthyroidism, Aliskiren, and Fosinopril.

	Control	Hyperthyroid	Aliskiren	Fosinopril
Urine flow	1.35±0.24	1.24±0.31	2.49±0.52	3.40±0.6
ml/hr	a	a	ab	b

Na^+^ excretion rate	44.81±9	52.42±16	138.75±26	175.23±40
mmol/hr	a	a	b	b

K^+^ excretion rate	.032±0.006	.01±0.001	.08±0.03	.09±0.02
mmol/hr	ab	a	bc	c

Different letters indicate significance differences at P<0.05.

Abbreviations. Na: sodium, K: potassium.

**Table 9:** Urine flow, Na^+^ excretion rate, and K^+^ excretion rate in hypothyroidism, Aliskiren, and Fosinopril.

	Control	Hypothyroid	Aliskiren	Fosinopril
Urine flow	1.35±0.24	1.24±0.28	2.07±0.44	.95±0.2
ml/hr	ab	ab	b	a

Na^+^ excretion rate	44.81±9	70.59±17	104.52±24	54.53±12
mmol/hr	a	ab	b	ab

K^+^ excretion rate	.03±0.006	.08±0.03	.04±0.007	.02±0.002
mmol/hr	ab	b	ab	a

Different letters indicate significance differences at P<0.05.

Abbreviations. Na: sodium, K: potassium.

**Table 10:** Levels of serum Na^+^ and K^+^ in hyperthyroid, hypothyroid, Aliskiren, and Fosinopril groups.

	Control	Hyperthyroid	Aliskiren	Fosinopril
Serum Na^+^	143.90±0.7	144.30±0.5	144.10±0.4	142.44±0.4
mmol/L	ab	b	b	a

Serum K^+^	4.72±0.4	5.17±0.2	5.49±0.5	4.79±0.07
mmol/L	a	a	a	a

	Control	Hypothyroid	Aliskiren	Fosinopril

Serum Na^+^	143.90±0.7	143.75±1	147.48±1	144.20±1
mmol/L	a	ab	b	ab

Serum K^+^	4.72±0.4	4.04±0.1	4.53±0.1	4.83±0.1
mmol/L	a	a	a	a

Different letters indicate significance differences at P<0.05.

Abbreviations. Na: sodium, K: potassium.

**Table 11:** Mean and SE (standard error) of weights of rats (in grams). Hyperthyroid, hypothyroid, and after treatment with Aliskiren and Fosinopril.

	Hyperthyroid	After treatment	P value
Aliskiren group	284± 14.00	272± 4.63	Ns
(Weight in grams)			

Fosinopril group	293± 10.90	280 ±2.73	Ns
(Weight in grams)			

	Hypothyroid	After treatment	

Aliskiren group	284± 7.00	272 ±4.00	Ns
(Weight in grams)			

Fosinopril group	293± 6.04	290±7.90	Ns
(Weight in grams)			

NS: nonsignificant.

## Data Availability

The data that support the findings of this study are included in the article and are available from the corresponding author.

## Abbreviations

BP: Blood pressure
RAAS: Renin-angiotensin-aldosterone system
PTU: Propylthiouracil
AST: Aspartate aminotransferase
ALT: Alanine transaminase
ALP: Alkaline phosphatase.

## Additional Points

Limitations of the Study. Lack of prior studies on the same topic was one of the major limitations of this study that lead to taking too many parameters in such a sample size. As this study is part of a Ph.D. study, there was limitation on the time dedicated to the research. We have come to find out that it would have been better for the study if it included normal rats, but because of time and funding limitations we were not able to perform this part of the study.
